# Predictive factors and prognosis of upper gastrointestinal bleeding in gastric cancer: A large population-based study (UGIB-GC trial)

**DOI:** 10.1371/journal.pone.0291926

**Published:** 2023-09-20

**Authors:** Anya Kiattiweerasak, Phubordee Bongkotvirawan, Natsuda Aumpan, Yoshio Yamaoka, Muhammad Miftahussurur, Ratha-korn Vilaichone

**Affiliations:** 1 Gastroenterology and Hepatology Center, Chulabhorn Hospital, Chulabhorn Royal Academy, Bangkok, Thailand; 2 Center of Excellence in Digestive Diseases and Gastroenterology Unit, Department of Medicine, Thammasat University, Pathumthani, Thailand; 3 Chulabhorn International College of Medicine (CICM) at Thammasat University, Pathumthani, Thailand; 4 Department of Environmental and Preventive Medicine, Faculty of Medicine, Oita University, Yufu, Japan; 5 Research Center for Global and Local Infectious Diseases, Oita University, Yufu, Japan; 6 Department of Medicine, Gastroenterology and Hepatology Section, Baylor College of Medicine, Houston, Texas, United States of America; 7 Division of Gastroentero-Hepatology, Department of Internal Medicine, Faculty of Medicine, Universitas Airlangga, Surabaya, Indonesia; National Healthcare Group, SINGAPORE

## Abstract

**Background:**

Gastric cancer remains the fourth leading cause of cancer-related death worldwide. Significant number of gastric cancer patients presented with bleeding.

**Objective:**

This study aimed to identify risk factors and overall survival rates of bleeding gastric cancer patients.

**Methods:**

This retrospective cohort study was conducted between 2007–2022 at tertiary care center in Thailand. Clinical information, endoscopic findings and histological type were extensively reviewed and were compared between bleeders and non-bleeders. Patients were monitored for at least 5 years.

**Results:**

There were 20,981 patients who underwent upper gastrointestinal endoscopy during study period. Total of 201 gastric cancer patients were included in this study, 21 were excluded due to incomplete medical records. 180 gastric cancer patients were included with mean age of 60.5±14.3 years. There were 65 (36.1%) patients with gastrointestinal bleeding. Hypertension and chronic kidney disease were significantly more common in bleeders than non-bleeders (43.1% vs 23.5%, OR2.51, 95%CI 1.14.-5.52, p = 0.022; and 16.9% vs 5.2%, OR2.00, 95%CI 1.56–6.63, p = 0.025, respectively). current *H*. *pylori* infection was also significantly more common in bleeders than non-bleeders (84.6% vs. 55.7%, OR 4.39, 95%CI 1.90–10.12, p<0.001). Median overall survival of bleeders was significantly lower than non-bleeders (7±0.93 vs 10±2.10 months, p = 0.001).

**Conclusions:**

Bleeding gastric cancer was not an uncommon condition. Majority of patients presented at advanced stage with grave prognosis. Male gender, hypertension, chronic kidney disease, and current *H*. *pylori* infection were reliable predictors for bleeding. Early diagnosis and prompt treatment are the key to improve clinical outcome.

## Introduction

Gastric cancer is the fourth leading cause of cancer-related death worldwide and remains a common gastrointestinal (GI) cancer in the Association of Southeast Asian Nations (ASEAN) [1]. Majority of malignant upper GI bleeding (58%) was caused by this particular cancer [2]. Gastric cancer patients presenting to the emergency department with upper GI bleeding mainly had an advanced unresectable stage resulting in higher mortality rates [3]. Apart from bleeding from friable tumor mucosa itself, *Helicobacter pylori* (*H*. *pylori*) infection is not only a group 1 carcinogen and strong risk factor for gastric cancer, but also associated with peptic ulcer disease causing GI bleeding [4, 5]. In addition, smoking, high salt diet, and high nitrite intake are also main risk factors for development of gastric cancer [5].

Bleeding from gastric cancer may cause severe anemia, hypovolemic shock, and life-threatening conditions resulting in death [6, 7]. These acute hemorrhagic events lead to treatment delays for patients requiring chemotherapy and complications from transfusion reactions. Therefore, achieving hemostasis is essential for improving the clinical outcome [8]. The American, European, and Japanese Guidelines for diagnosis and management of non-variceal upper GI bleeding recommend early upper GI endoscopy and endoscopic therapy following prompt hemodynamic resuscitation [9–11]. However, more information about risk factors and severity of bleeding is required to offer appropriate management for bleeding in gastric cancer [2, 12, 13].

Until now, there have been limited number of studies comparing survival rates of patients with bleeding gastric cancer to those of non-bleeders especially in ASEAN. This study would be a large population-based and pioneer study emphasizing on this important cancer, identifying risk factors and overall survival rates that could lead to appropriate management of bleeding gastric cancer in ASEAN.

## Material and methods

### Patients and study design

This retrospective cohort study enrolled all patients with gastric cancer at tertiary care center in Thailand between August 2007 and August 2022. Gastric cancer was diagnosed by histopathology of optimal gastric biopsies. Demographic data, clinical presentations, histopathology, cancer staging, current status of *H*. *pylori* infection, laboratory results, endoscopic findings, treatment options, and clinical outcomes were extracted from medical records and extensively reviewed. Patients with incomplete medical record were excluded from this study.

### Definitions

**Upper gastrointestinal bleeding** was defined as patients presenting with hematemesis, coffee-ground vomiting, melena, or hematochezia.

**Dyspepsia** was defined as having one or more bothersome symptoms of epigastric pain, burning, postprandial fullness, or early satiation [14].

**History of alcohol drinking** was defined as alcohol drinking more than 5 gm per day [15].

**History of smoking** was defined as smoking more than 10 cigarettes per day [16].

**Early-stage gastric cancer** was defined as primary gastric cancer with the invasion depth no further than submucosa regardless of lymph node involvement. According to the American Joint Committee on Cancer TNM staging, early gastric cancer is defined as T1 with any N [17].

**Advanced stage gastric cancer** was defined as primary gastric cancer with the invasion depth further than submucosa. According to the American Joint Committee on Cancer TNM staging, advanced gastric cancer is defined as gastric cancer with tumor size greater than T1 with any N [17].

**Current *H*. *pylori* infection** was defined as presence of *H*. *pylori* in histopathology of gastric biopsies, positive rapid urease test, or positive culture. Majorities of our patients underwent more than two methods to ensure the most accurate assessment of the current prevalence of *H*. *pylori* infection.

**Rebleeding** was defined as recurrent bleeding or a decrease in hemoglobin concentration > 2 g/dL within 24 hours after initial hemostasis.

**Anemia** was defined as hemoglobin concentration <12 g/dL in non-pregnant women or <13 g/dL in men according to the criteria from World Health Organization (WHO) [18].

**Thrombocytopenia** was defined as platelet count of less than 150 x 10^9^/L.

### Statistical analysis

Demographic data were analyzed by Pearson’s chi-squared test or Fisher’s exact test whichever appropriate. Factors associated with one-year mortality were analyzed by using cox multivariate analysis and factors associated with bleeding gastric were analyzed by logistic regression. In our study, univariate analysis was performed to identify significant demographic or other risk factors associated with bleeding gastric cancer and one-year mortality. Every variable with a p-value of less than 0.05 by univariate analysis were entered into the multivariate analysis and backward elimination method were used to identify independent predictors of bleeding gastric cancer. Kaplan-Meier survival functions with corresponding estimates and 95% CI for survival were calculated. All tests were two-sided and p-value of <0.05 was considered as statistical significance. The statistical analysis was performed by using SPSS version 27 (SPSS Inc, Chicago, Illinois) and STATA statistical software version 18 (StataCorp. 2023. *Stata Statistical Software*: *Release 18*. College Station, TX: StataCorp LLC). The study protocol was approved by the Human Research Ethics Committee of Thammasat University No.1 (Faculty of Medicine) with number of approval number of 155/2563and was conducted according to the good clinical practice guideline, as well as the 1975 Declaration of Helsinki. As this study was conducted retrospectively, the Ethics Committee had waived the requirement for informed consent to access patient data, and all data were analyzed anonymously.

## Results

There were 20,981 patients underwent upper gastrointestinal endoscopy during study period. A total of 201 gastric cancer patients were included in this study. The prevalence of gastric cancer from this study was 0.96%. However, 21 were excluded due to incomplete medical records. Of 180 patients with gastric cancer, 88 (48.9%) were males with the mean age of 60.5±14.3 years. There were 65 (36.1%) patients with upper GI bleeding. Hypertension (30.6%), dyslipidemia (21.7%) and diabetes mellitus (16.7%) were common comorbidities. Weight loss (70%) and dyspepsia (59.4%) were also common presenting symptoms. The prevalence of diffuse-type gastric cancer was comparable to the intestinal type (51.7% vs. 48.3%). Most gastric cancer patients were at advanced stage (93.3%) with distant metastases (65%). Baseline characteristics and endoscopic findings of gastric cancer patients were demonstrated in Tables 1 and 2.

**Table 1:** Baseline characteristics of all gastric cancer patients.

Characteristics	Bleeding GC (N = 65)	Non-bleeding GC (N = 115)	P-value
Age (years ± SD)	59.23±13.77	60.91±15.05	0.919
Male	39 (60%)	49 (42.6%)	0.025
**Comorbidities**			
Hypertension	28 (43.1%)	27 (23.5%)	0.006
Dyslipidemia	15 (23.0%)	24 (20.9%)	0.730
Diabetes mellitus	14 (21.5%)	16 (13.9%)	0.187
Chronic kidney disease	11 (16.9%)	6 (5.2%)	0.010
Cirrhosis	5 (7.8%)	1 (0.9%)	0.024
Multiple comorbidities	27 (41.5%)	27 (23.5%)	0.011
Antiplatelet use	11 (16.9%)	10 (8.7%)	0.099
Anticoagulant use	2 (3.1%)	2 (1.7%)	0.621
Smoking	27 (41.5%)	29 (25.2%)	0.023
Alcohol drinking	32 (49.2%)	33 (28.7%)	0.006
Current *H*. *pylori* infection	55 (84.6%)	64 (55.7%)	<0.001
**Clinical manifestations**			
Weight loss	43 (66.2%)	83 (72.2%)	0.397
Dyspepsia	34 (52.3%)	73 (63.5%)	0.143
Anemia	34 (52.3%)	29 (25.2%)	<0.001
Loss of appetite	28 (43.1%)	60 (52.2%)	0.241
Nausea and vomiting	13 (20.0%)	41 (35.6%)	0.028
Abdominal pain	10 (15.4%)	40 (34.8%)	0.005
Thrombocytopenia	38 (58.5%)	26 (22.6%)	<0.001
**Location**			
Cardia	16 (24.6%)	32 (28.8%)	0.682
Non-cardia	49 (75.4%)	83 (72.2%)	
**Histological types**			
Intestinal type	34 (52.3%)	53 (46.1%)	0.422
Diffuse type	31 (47.7%)	62 (53.9%)	
**Cancer staging**			
Early stage	4 (6.2%)	8 (7%)	1.00
Advanced stage	61 (93.8%)	107 (93%)	
**Treatment**			
Surgery	27 (41.5%)	45 (39.1%)	0.751
Chemotherapy	33 (51.6%)	60 (52.2%)	0.856
Concurrent chemoradiotherapy	3 (4.7%)	8 (7.0%)	0.748
Targeted therapy	2 (3.1%)	2 (1.7%)	0.621

**Table 2:** Endoscopic finding in patients with gastric cancer.

Endoscopic findings	Bleeding GC (N = 65)	Non-bleeding GC (N = 115)	P-value
**Related to GC**			
Mass	26 (44.1%)	55 (47.8%)	0.286
Ulcer	18 (30.5%)	23 (20%)	0.237
Ulceroproliferative mass	9 (15.3%)	19 (16.5%)	0.634
Linitis plastica	8 (13.6%)	18 (15.7%)	0.540

Regarding treatment options, 93 patients received chemotherapy for palliative treatment (51.7%). 72 (40%) patients underwent surgery. 11 (6.1%) had chemoradiotherapy, and four (2.2%) received targeted therapy.

### Predictive factors for bleeding gastric cancer

The results of the univariate analysis revealed that male gender, smoking, history of alcohol drinking and smoking, hypertension, chronic kidney disease, abdominal pain, anemia, thrombocytopenia, and current *H*. *pylori* infection were associated with bleeding gastric cancer. To determine the factors used in the subsequent multivariate analysis, all significant factors in univariate analysis were considered.

Multivariate analysis demonstrated that men, hypertension and chronic kidney disease were significantly more common in bleeding gastric cancer than non-bleeders (60% vs 42.6%, OR1.92, 95%CI1.01–3.67, p = 0.047; 43.1% vs 23.5%, OR2.51, 95%CI 1.14.-5.52., p = 0.022; and 16.9% vs 5.2%, OR2.00, 95%CI 1.56–6.63, p = 0.025 respectively). Interestingly, abdominal pain was less common in bleeders than non-bleeders (15.4% vs 34.8%, OR 0.31, 95%CI 0.13–0.74, p = 0.008). current *H*. *pylori* infection were significantly more common in bleeding cancer than non-bleeders (84.6% vs. 55.7%, OR 4.39., 95%CI 1.90–10.12, p<0.001). Detailed univariate and multivariate analysis of factors associated with bleeding gastric cancer was also demonstrated in Table 3.

**Table 3:** Univariate and multivariate analysis of factors associated with bleeding gastric cancer.

Variables	Univariate analysis			Multivariate analysis		
	OR	(95% CI)	p-value	OR	(95% CI)	p-value
Male	2.02	(1.08–3.75)	0.026	1.92	(1.01–3.67)	0.047
Age ≥ 60-year-old	0.81	(0.44–1.50)	0.511			
Smoking	2.11	(1.10–4.03)	0.024	0.95	(0.40–2.26)	0.903
Alcohol	2.41	(1.28–4.53)	0.006	2.26	(0.96–5.36)	0.063
Hypertension	2.46	(1.28–4.74)	0.007	2.51	(1.14–5.52)	0.022
Chronic kidney disease	3.70	(1.30–10.54)	0.014	2.00	(1.56–6.63)	0.025
Cirrhosis	9.50	(1.09–83.1)	0.042	2.21	(0.22–22.3)	0.503
Current *H*. *pylori* infection	4.38	(2.03–9.44)	<0.001	4.39	(1.90–10.12)	<0.001
Abdominal pain	0.34	(0.16–0.74)	0.007	0.31	(0.13–0.74)	0.008

### Hemostatic intervention in bleeding gastric cancer

Of 65 patients with bleeding gastric cancer, 12 (18.5%) needed hemostatic intervention including hemoclip application with or without adrenaline injection and argon plasma coagulation (APC) as demonstrated in Table 4. Nearly all patients successfully achieved hemostatic intervention except for three patients. The first patient required angiography with embolization. The second patient underwent surgical treatment to stop massive upper GI bleeding and the third patient received combination of hemoclip application with adrenaline injection for treatment of rebleeding condition.

**Table 4:** Hemostatic intervention of bleeding gastric cancer.

**Endoscopic management**	**(N = 10)**
Hemoclip application	3 (30%)
Argon plasma coagulation (APC)	1 (10%)
Bipolar coaptation	1 (10%)
Hemostatic powder (TC-325)	1 (10%)
**Combination of hemostatic procedures**	4 (40%)
Hemoclip application + APC	2 (20%)
Hemoclip application + Adrenaline injection	2 (20%)
**Non-endoscopic management**	**(N = 2)**
Surgery	1 (10%)
Angiography with embolization	1 (10%)

### Survival outcomes of bleeding and non-bleeding gastric cancer patients and predictive factors associated with 1-year mortality

There were 165 patients coming for follow-up visits, whereas 15 who lost to follow-up were excluded from the survival analysis. The overall 1-year and 2-year survival rates were 33.9% and 12.7%. 56 out of 150 patients died in first year, with 51 deaths attributed to cancer-related causes, three to infection, one to acute coronary syndrome, and one to massive pulmonary embolism. In the two-year survival analysis, 21 patients died, with 16 deaths attributed to cancer-related causes and five to infection.

**Fig 1:**
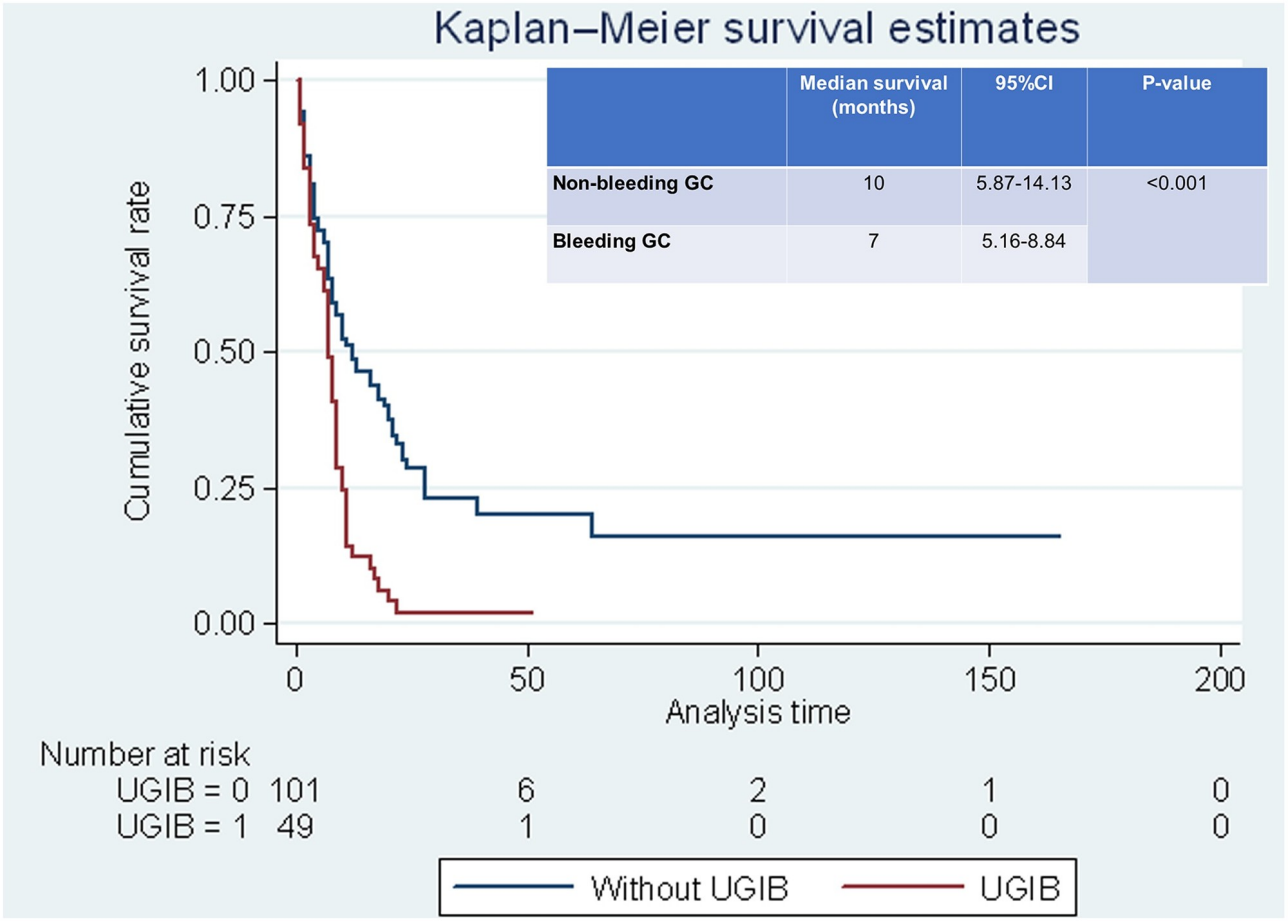
Survival function analysis: Bleeding GC vs. non-bleeding GC.

Furthermore, in survival analysis the median overall survival of bleeding gastric cancer patients was significantly lower than the non-bleeding group (7±0.93 months vs 10±2.10 months, p<0.001) as demonstrated in Fig 1.

In the cox regression analysis conducted to determine factors associated with 1-year mortality in gastric cancer patients, univariate analysis revealed that upper GI bleeding was the only factor with statistically significantly associated with one-year mortality (OR 2.15, 95%CI; 1.50–3.09. p-value < 0.001). When multivariate analysis was performed, taking into account factors known to be associated with mortality such as male gender, age ≥ 60 years, and advanced stage cancer, upper GI bleeding remained significantly associated with 1-year mortality (OR 2.30, 95%CI; 1.56–3.41. p-value < 0.001). Table 5 demonstrated the results of both univariate and multivariate Cox regression analysis of factors associated with 1-year mortality.

**Table 5:** Univariate and multivariate cox regression analysis of factors associated with 1-year mortality.

Variables	Univariate analysis			Multivariate analysis		
	OR	(95% CI)	p-value	OR	(95% CI)	p-value
Male	1.09	(0.78–1.54)	0.611	0.98	(0.69–1.40)	0.925
Age ≥ 60-years old	0.83	(0.59–1.18)	0.293	0.89	(0.62–1.27	0.512
Smoking	1.09	(0.76–1.57)	0.643			
Alcohol	1.19	(0.83–1.69)	0.341			
Diabetes mellitus	1.21	(0.76–1.91)	0.420			
Hypertension	1.14	(0.76–1.63)	0.577			
Chronic kidney disease	0.88	(0.48–1.63)	0.688			
Cirrhosis	1.23	(0.50–3.00)	0.651			
Anemia	1.21	(0.84–1.75)	0.304			
Thrombocytopenia	1.08	(0.76–1.54)	0.671			
Upper GI bleeding	2.15	(1.50–3.09)	<0.001	2.30	(1.56–3.41)	<0.001
Current *H*. *pylori* infection	1.13	(0.77–1.65)	0.521			
Advanced stage	1.96	(0.86–4.46)	0.108	2.45	(0.99–6.13)	0.052

## Discussion

Gastric cancer is accountable for almost 800,000 deaths annually [1, 19]. Upper GI bleeding was a presenting symptom in most of gastric cancer patients with an advanced stage [3]. This study revealed that majority of participants were men in elderly age, diagnosed at advanced stage and mostly had intestinal type of gastric cancer which were similar to prior studies [8, 20]. Our study also indicated that hypertension was an important predictive factor for bleeding condition. The explanation might be from shear stress-induced mechanotransduction that further induces nitric oxide production resulting in inhibition of platelet aggregation and thrombus formation [21, 22]. chronic kidney disease was also associated with upper GI bleeding condition in previous study [23]. Use of anticoagulation during hemodialysis and platelet dysfunction might be accounted for higher risk of upper GI bleeding [24]. Current *H*. *pylori* infection induces inflammation in mucosal layer contributing to epithelial cell degeneration and injury, defect in mucosal protection, and eventually causes higher risk of upper GI bleeding in gastric cancer [25]. Previous research has demonstrated that levels of pro-inflammatory cytokines, such as interleukin IL-1β, IL-6, and IL-8, are increased in gastric mucosa infected with *H*. *pylori*, in contrast to uninfected normal-appearing gastric mucosa. Furthermore, studies have demonstrated that levels of IL-1β, IL-6, and IL-8 in gastric cancer tissues are significantly elevated in *H*. *pylori*-positive cases compared to *H*. *pylori*-negative cases. These findings indicate that *H*. *pylori* infection can contribute to the development of gastric cancer by inducing chronic inflammation and potentially influencing cancer bleeding [26]. Thrombocytopenia observed in gastric cancer can be elucidated through a various factors. Among these factors are bone marrow metastasis, chemotherapy-induced thrombocytopenia, consumptive coagulopathy, and in rare cases, autoimmune thrombocytopenia [27]. However, it should be noted that anemia and thrombocytopenia should be coincidence of bleeding gastric cancer and might not be counted as predictive factors.

Abdominal pain as clinical presentation was inversely associated with bleeding cancer in our study. Gastric cancer presented with abdominal pain might have a better chance to be promptly investigated and early detected leading to appropriate treatment before the occurrence of bleeding in the future. A previous study suggested that upper GI bleeding was related to the use of anti-platelet drugs such as low-dose aspirin [28]. However, we could not demonstrate any association between taking antiplatelet or anticoagulant medications and bleeding condition. This disparity may have been caused by concurrent use of anti-secretary agents, e.g., proton-pump inhibitor (PPI). Cancer location, histological subtype, and staging were rarely reported to be associated with bleeding gastric cancer [8, 13]. Identifying gastric cancer bleeding risk factors can have significant implications for clinical practice. Minimizing these risk factors such as *H*. *pylori* eradication, blood pressure control and reduce further kidney injury could be important to reduce upper GI bleeding risk in such patients.

Most bleeding gastric cancer patients achieved hemostasis by endoscopic intervention with multi-modality approach including APC, adrenaline injection, bipolar coaptation, and hemoclip application [13]. Human-derived fibrin glue (Beriplast) injection and hemostatic agent TC-325 (Hemospray^®^) might be new important strategies to assist physicians to achieve successful management in massive and clinical unstable upper GI bleeding in gastric cancer [8, 29]. The median survival of bleeding gastric cancer patients in our study was higher than previous reports which might be from higher success of hemostasis by multimodality of endoscopic techniques and prompt resuscitation in critically ill patients by current practice in ASEAN that led to prolonged survival of this special group of patients in the past decades [13, 30].

In conclusion, gastric cancer is a common GI cancer and a major contributor to cancer-related deaths in ASEAN and globally. A significant number of gastric cancer patients presented with overt upper GI bleeding. Male gender, hypertension, chronic kidney disease, and current *H*. *pylori* infection were predictors for bleeding gastric cancer. Early diagnosis, high suspicion in high-risk groups and promptly appropriate management are crucial and will definitely improve treatment outcomes.

## Supporting information

S1 Appendix PLOS’ questionnaire on inclusivity in global research. (DOCX)
